# Management of Post-orthodontic White Spot Lesions and Subsequent Enamel Discoloration with Two Microabrasion Techniques

**Published:** 2015-03

**Authors:** Arezoo Jahanbin, Hamideh Ameri, Mostafa Shahabi, Ala Ghazi

**Affiliations:** 1 Dept. of Orthodontics, Dental Research Centre, School of Dentistry, Mashhad University of Medical Sciences, Mashhad, Iran;; 2 Dept. of Restorative Dentistry, Dental Research Centre, School of Dentistry, Mashhad University of Medical Sciences, Mashhad, Iran;; 3 Dept. of Oral Medicine, School of Dentistry, Mashhad University of Medical Sciences, Mashhad, Iran;

**Keywords:** White Spot Lesion, Microabrasion, Discoloration, Orthodontics

## Abstract

**Statement of the Problem:**

Demineralization of enamel adjacent to orthodontic appliances frequently occurs, commonly due to insufficient oral hygiene.

**Purpose:**

The aim of this study was to compare two microabrasion techniques on improving the white spot lesions as well as subsequent enamel discoloration.

**Materials and Method:**

Sixty extracted premolar teeth without caries and hypoplasia were selected for this study. White spot lesions were artificially induced on the buccal surface of each tooth. Teeth were randomly assigned to three treatment groups, each treated with pumice powder as the control, microabrasion with 18% HCl, and microabrasion with 37% H_3_PO_4_. Subsequently, the three groups were daily immersed for five minutes in a tea-coffee solution for a period of one week. Colorimetric evaluation was done before and after formation of white spot lesions, after microabrasion, and after immersion in the colored solution; then the color differences (∆E) were calculated. Statistical analysis was performed by multiple measurement analysis and the Tukey’s test.

**Results:**

This study showed that ∆E between the stages of white spot formation and microabrasion for H_3_PO_4_ was more than other groups and for the pumice powder group it was less than the others. Furthermore, there was a significant difference between ∆E of the three study groups (*p*= 0.017). Additionally, ∆E after placing the teeth in the colored solution and microabrasion was the highest for the HCl group and the lowest for the pumice powder group. There was also a significant difference between the three groups (*p*= 0.000).

**Conclusion:**

Pumice powder alone had similar effects as 18% HCl on removing the white spot lesions. Nevertheless, 18% HCl makes the enamel susceptible for subsequent color staining more than the other microabrasion methods.

## Introduction

White spot lesions are subsurface enamel porosities caused by enamel demineralization.[1] According to Ogaard et al. white spot lesions are the most prevalent iatrogenic side effect of orthodontic treatment. These lesions develop as a result of prolonged plaque accumulation on the enamel surfaces adjacent to orthodontic devices, commonly due to poor oral hygiene.[2-3]

After active orthodontic treatment, demineralization process is normally expected to decelerate due to alterations in these local factors. Some white spot lesions may sometimes remineralize and return to normal or at least to a visually acceptable appearance. However, some white spot lesions persist and become unsightly. In severe cases, white spot lesions may progress to enamel carious lesions which require restorative  treatment. Hence, early diagnosis and treatment of these lesions by dentists would be of particular importance.[4]

Various treatments have been proposed to improve the appearance of white spot lesions including restorative procedures, improvement of remineralization using high concentrations of topical fluoride or casein phosphopeptide amorphous calcium phosphate (CPP-ACP), chewing gum to promote remineralization, microabrasion, and argon-laser irradiation.[5-6]

Microabrasion technique involves application of hydrochloric acid and pumice in form of a paste, to the affected enamel surfaces, which removes about 100µ of the enamel through a combination of erosion and  abrasion. In this technique, polished enamel does not have the typical enamel surface appearance because no interprismatic space exists in microabraded enamel. This highly polished enamel surface, compared with intact natural enamel, is more resistant to bacterial colonization and demineralization. Therefore, this technique would not place the patient at risk of demineralization and its progression.[7-8] In this regard, Willis et al. concluded that microabrasion with hydrochloric acid is an appropriate technique to employ prior to initiating more aggressive cosmetic dentistry.[9]

Another study has shown that the application of microabrasion technique with hydrochloric acid and pumice resulted in 83% reduction in the size of white spot lesions after the treatment. Therefore, the researchers concluded that microabrasion technique is an effective treatment for the improvement of long-standing post-orthodontic demineralized enamel lesions.[10]

Furthermore, Pliska et al. examined the effects of application of CPP-ACP paste and microabrasion treatment on the regression of artificially-induced white spot lesions in bovine enamel. They demonstrated that microabrasion treatment with or without CPP-ACP reduce white spot lesions.[11]

Previously, Meireles et al. in 2009 evaluated the surface roughness and enamel loss resulting from two microabrasion methods. Evaluation of samples by a stereomicroscope (40X) and the image tool software revealed that the mean surface roughness by hydrochloric acid was statistically lower than phosphoric acid (*p*< 0.001). Deeper demineralization depth (*p*< 0.003) and a larger total demineralization area were observed in teeth treated with hydrochloric acid as compared with those treated with phosphoric acid (*p*< 0.005). This study showed that application of phosphoric acid is safer and more convenient to perform.[12]

The effects of the two microabrasion methods - using 18% hydrochloric acid and 37% phosphoric acid on improvement of post-orthodontic white spot lesions and subsequent enamel discoloration has not been investigated yet, therefore, our study aims on comparison of these two techniques. 

## Materials and Method

Ethics approval was obtained from the Ethics Committee of Mashhad University of Medical Sciences prior to the beginning of this research project.

Sixty upper premolars, extracted for orthodontic purposes, were selected to be used in this study. Only premolars free of damage from extraction forceps and with no evidence of enamel hypoplasia or enamel defects were used. The teeth were cleaned and stored in normal saline until required. The teeth were cleaned and polished with prophylactic rubber cups for 5 seconds and then thoroughly washed and dried. The teeth were randomly assigned to three equal groups of twenty each.

*Baseline colorimetric evaluation*

At baseline, color measurement for the buccal surface of all teeth was recorded with a colorimeter (Color Eye XTH; Gretagm Macbeth, USA) which recorded the Commission Internationale de l’Eclairage Lab values (CIElab). The L-value corresponds to the degree of lightness in the Munsell system, whereas the a-values and the b-values, respectively, give the position on red or green (+a = red, -a = green) and yellow or blue (+b= yellow,-b=blue) axes.

Palatal sides of the teeth were embedded in a mold of Speedex (Colten; Asia Chemi Teb Co., Tehran, Iran) in order to fix the specimens during colorimetery. The colorimeter was automatically calibrated before each precision. All measurements were done by keeping the colorimeter perpendicular and flush to the buccal surface of the tooth, and in contact with the buccal tooth surface. The color was evaluated from the middle of the buccal teeth surfaces. Measurements were repeated two times for each specimen and the mean values of data were calculated. To minimize the effect of ambient light on the results, all colorimetric evaluations were carried out in the same examination room in sunny days between 11 a.m. to 13 p.m. with standardized lighting conditions. 

*White Spot Formation*

The specimens were kept individually in a demineralizing solution (2.0 mM calcium, 2.0 mM phosphate, 0.030 ppm F, in 75 mM acetate buffer, pH 4.3) for 3 hours (20 ml per block), and in a remineralizing solution (1.5 mM calcium, 0.9 mM phosphate, 150 mM of KCl, 0.050 ppm F in 20 mM cacodylic buffer, pH 7.4) for 20 hours (10 ml per block) each day. After each cycle, the specimens were returned to the same solutions. This cycle was repeated daily for 5 days and the teeth remained in the remineralizing solution for 2 days. The experiment was carried out at 37ºC.[13]

*Second colorimetery*

The second colorimetery (after white spot formation) was performed exactly as the baseline colorimetery and L, a, b values were recorded. Changes in the color of each specimen in comparison with the initial colorimetery results (∆E1) were calculated according to the following formula:

$$∆E=\sqrt{{\left(∆a\right)}^{2}+{\left(∆b\right)}^{2}+{\left(∆l\right)}^{2}}$$

*White spot elimination*

The first group was polished with pumice powder. Microabrasion technique with 18% hydrochloric acid, and 37% phosphoric acid was performed for the second and the third groups, respectively. For all groups, the treatment was done using a prophylactic rubber cap with slight pressure for five seconds.[12] After this stage, L, a, b were again measured and the color differences were compared with the second colorimetery (∆E2).

*Staining Stage*

The teeth were immersed in a tea-coffee solution for one week. This solution was prepared as follows: 1 g of tea and 1 g of coffee were mixed in 100 cc of boiled water and then filtered. The teeth were placed in the solution daily for five minutes. Subsequent to this stage, the L, a, b values were determined again and color differences were compared with the second colorimetery (∆E3). The teeth from all groups were preserved in Fusayama-Meyer artificial saliva at pH of 7.3 containing KCI (0.4 g/l), NaCl (0.4 g/l), CaCl_2_, 2H_2_O (0.906 g/l), NaH_2_PO_4_, 2H_2_O (0.690 g/l), Na_2_S, 9H_2_O (0.005 g/l), Urea (1 g/l), and distilled water at 37°C.

*Statistical analysis*

The Data were analyzed by SPSS (version 10.0) and statistical analysis was performed by using the multiple measurement analysis. Tukey’s test was done for pair-wise comparison between the means. The significant level was set at *p*< 0.05.

## Results

Based on multiple measurement test, there was an interaction effect between the groups and between different stages (*p*< 0.05). Furthermore, at an error level of 5%, there were significant differences in ∆E values of all study groups (*p*< 0.05).

The means and standard deviations of ∆E for the three groups have been shown in Table 1. According to this table, no significant difference was observed between ∆E1 of all groups under study (*p*= 0.106).

**Table 1:** Mean±SD of ∆E and results of statistical analysis in the three study groups in three stages colorimetery

**∆E**	**N**	**Mean**	**S.D**	**P-value**
∆E1				0.106
Pumice powder	20	5.71	3.05	
HCl	20	4.08	1.74	
H3PO4	20	4.33	2.73	
∆E2				
Pumice powder	20	6.80	3.17	0.017
HCl	20	8.74	2.53	
H3PO4	20	9.46	3.06	
∆E3				
Pumice powder	20	10.22	4.59	0.000
HCl	20	19.41	4.23	
H3PO4	20	15.62	5.16	

As shown in Table 2, the mean ∆E2 for the 37% phosphoric acid group was higher than the 18% hydrochloric acid group, while it showed the lowest value for the pumice powder (control) group. Furthermore, there was a significant difference between ∆E2s of the three study groups (*p*= 0.017).

**Table 2:** Results of Tukey’s test for comparison of ∆E2 in the three study groups

**Group**	**N**	**Subset for Alpha=0.05**	
		**1**	**2**
Pumice powder	20	6.8068	8.7499
HCl	20	8.7499	
H3PO4	20	9.4617	0.725
Sig		0.101	

Additionally, ∆E3, which was the mean difference between L, a, b after placing the teeth in the color solution and L, a, b after microabrasion, was the highest for the HCL group and the lowest for the pumice powder group. There was also a significant difference between ∆E3s of the three groups (*p*= 0.000) (Table 3).

**Table 3:** Results of Tukey’s test for comparison of ∆E3 in the three study groups

**Group**	**N**	**Subset for Alpha=0.05**		
		**1**	**2**	**3**
Pumice powder	20	10.2215		
H3PO4	20		15.6219	
HCl	20			19.4125
Sig		1.000	1.000	1.000

## Discussion

Despite the previous efforts made by many researchers to prevent white spot formation around the fixed orthodontic appliances, this problem is still not resolved. However, most of the efforts have been focused on prevention of white spot formation rather than treatment of these lesions. The objective of the conservative treatments is based on demineralization of subsurface lesions. As a general procedure for treatment of white spot lesions, initially the most conservative methods such as topical fluoride should be performed. If white spot lesions still remain after this procedure, microabrasion is the treatment of choice.[14]

Willmote examined the effect of fluoride and saliva on treatment of white spot lesions after removal of fixed orthodontic appliances. He observed that the sizes of the post-orthodontic demineralized white spot lesions were reduced within six months following the treatment to approximately half the original size; however, the lesions were not completely removed.[15] Furthermore, Ogaard et al. reported that the application of concentrated fluoride agents for treating white spot lesions would arrest the lesion and prevent complete repair. In particular, deep lesions tend to remineralize only superficially while the underlying lesion body still remains porous.[2]

Enamel microabrasion is a method of treatment for color elimination, improving surface texture and remineralization.[16] Son et al. compared microabrasion with resin infiltration technique in a clinical study and reported that the resin infiltration technique was more effective for the treatment of white spot lesions.[5] Moreover, it has been shown that enamel microabrasion is a conservative method to eliminate enamel staining. The application of this method in addition to saving time, if used correctly, will improve teeth appearance without requiring mechanical tooth preparation, pain, and sensitivity.[7]

In the present study, we employed two microabrasion techniques to remove white spot lesions. Based on the results of this study, microabrasion with pumice powder was as effective as that with 18% hydrochloric acid and pumice powder in removing white spot lesions. However, in a study by Meireles et al., it was reported that both hydrochloric and phosphoric acids effectively removed these lesions.[12] Furthermore, Murphy et al. showed that the microabrasion method by using 18% hydrochloride acid and pumice was an effective approach for the improvement of long-standing post-orthodontic demineralized enamel lesions.[10] Also, it has been revealed that the microabrasion technique using 18% hydrochloride acid, pumice and glycerin removes completely all mild lesions and improves severe lesion to an appreciable extent.[17] Same findings have also been reported in a study by Bezerra et al.[18]

In the current study, after placing the teeth in a tea-coffee solution, the hydrochloric acid and the pumice powder groups showed the most and the least color changes, respectively. This result, as it has been suggested by Meireles et al., may be due to a deeper demineralization size after hydrochloric acid application.[12] Color differences greater than two units might be recognized, but most clinical studies set the limit for a visible color change at 3.7 units. The current study used the color difference of 3.7 units as a threshold between an acceptable and unacceptable color change.[19] Furthermore, the calculated color changes in all the stages of assessment and in all groups were higher than 3.7 units that could be recognized by the naked eye.

Finally, it must be noted that the using staining solution, immersion time, dilution with saliva and whether the teeth are cleaned or not during the study would be some of the factors that may determine the severity of enamel staining. 

## Conclusion

Despite the limitations of this study, microabrasion with pumice powder alone had similar effects as with 18% hydrochloric acid in removing the white spot lesions. Nevertheless, 18% hydrochloric acid would make the enamel more susceptible to subsequent color staining in comparison with the other microabrasion methods. 
